# Brain transcriptomics reveal the activation of neuroinflammation pathways during acute *Orientia tsutsugamushi* infection in mice

**DOI:** 10.3389/fimmu.2023.1194881

**Published:** 2023-06-22

**Authors:** Yuejin Liang, Aditi, Florence Onyoni, Hui Wang, Casey Gonzales, Piyanate Sunyakumthorn, Ping Wu, Parimal Samir, Lynn Soong

**Affiliations:** ^1^ Department of Microbiology and Immunology, University of Texas Medical Branch, Galveston, TX, United States; ^2^ Institute for Human Infections and Immunity, University of Texas Medical Branch, Galveston, TX, United States; ^3^ Department of Pathology, University of Texas Medical Branch, Galveston, TX, United States; ^4^ Department of Veterinary Medicine, United States Army Medical Directorate, Armed Forces Research Institute of Medical Sciences (United States MD-AFRIMS), Bangkok, Thailand; ^5^ Department of Neuroscience, Cell Biology and Anatomy, University of Texas Medical Branch, Galveston, TX, United States; ^6^ Department of Biochemistry and Molecular Biology, University of Texas Medical Branch, Galveston, TX, United States

**Keywords:** neuroinflammation, scrub typhus, IFN response, microglial activation, BBB disruption, innate immune activation, RNA-Seq - RNA sequencing

## Abstract

Scrub typhus, an acute febrile illness caused by *Orientia tsutsugamushi* (*Ot*), is prevalent in endemic areas with one million new cases annually. Clinical observations suggest central nervous system (CNS) involvement in severe scrub typhus cases. Acute encephalitis syndrome (AES) associated with *Ot* infection is a major public health problem; however, the underlying mechanisms of neurological disorder remain poorly understood. By using a well-established murine model of severe scrub typhus and brain RNA-seq, we studied the brain transcriptome dynamics and identified the activated neuroinflammation pathways. Our data indicated a strong enrichment of several immune signaling and inflammation-related pathways at the onset of disease and prior to host death. The strongest upregulation of expression included genes involved in interferon (IFN) responses, defense response to bacteria, immunoglobulin-mediated immunity, IL-6/JAK-STAT signaling, and TNF signaling via NF-κB. We also found a significant increase in the expression of core genes related to blood-brain barrier (BBB) disruption and dysregulation in severe *Ot* infection. Brain tissue immunostaining and *in vitro* infection of microglia revealed microglial activation and proinflammatory cytokine production, suggesting a crucial role of microglia in neuroinflammation during scrub typhus. This study provides new insights into neuroinflammation in scrub typhus, highlighting the impact of excessive IFN responses, microglial activation, and BBB dysregulation on disease pathogenesis.

## Introduction

Scrub typhus is a mite-borne infection caused by *Orientia tsutsugamushi* (*Ot*), an obligate intracellular bacterium closely related to *Rickettsia*. This disease causes at least one million clinical cases per year in the “tsutsugamushi triangle,” a region encompassing much of northern and eastern Asia, islands of the western Pacific Ocean, and a portion of northern Australia (1) However, new cases have been reported in other geographic areas, including South America (2). Yet, no vaccines are currently available for scrub typhus. Delayed antibiotic treatment is common due to non-specific symptoms at the initial stages of infection and missed or delayed diagnosis (3). In some patients, the bacteria spread systemically and cause severe outcomes, including interstitial pneumonia, myocardial and hepatic lesions, meningoencephalitis, acute respiratory distress syndrome, and multi-organ failure. The mortality rates can range from 0% to 70%, with a median of 6%, if left untreated (4).

The central nervous system (CNS) involvement is common among severe cases of scrub typhus with diverse neurological symptoms (headache, vomiting, altered sensorium, and seizures), but this clinical experience has largely been overlooked (5–8). The epidemiological studies in India and Thailand have suggested that 15%-25% of patients with scrub typhus had neurological complications (9–11). Importantly, CNS infections are positively correlated with mortality in scrub typhus (12). One meta-analysis study has illustrated that around 4% of patients with neurological manifestations of scrub typhus die in hospitals, and that case fatality rate may be higher in children than in adults (13, 14). Another clinical study has also indicated that most patients who recovered from acute encephalitis syndrome (AES) caused by *Ot*, often experience cognitive and behavioral impairments, and that more than 50% of them exhibited mild to severe degree of disability, raising a concern of long-term care requirement (15). A more recent population-based cohort study in Korea revealed that a previous episode of scrub typhus infection during old age is significantly linked to a higher risk of developing dementia, particularly Alzheimer’s disease (16), suggesting that *Ot*-triggered neuroinflammation may contribute to the pathogenesis of neurodegeneration.

The mechanisms of CNS disorders in scrub typhus remain unclear. Emerging evidence from animal studies suggests that excessive immune responses could be a potential contributor to the development of neuroinflammation and short- and long-term CNS damage (17). It is known that cytokines play an important role in both the normal development of the brain and in brain injuries. T helper 1 (Th1) cell cytokines, such as IFN-γ and TNF-α are considered inflammatory mediator in the brain following infection or injury, while Th2 cytokines (IL-4, IL-5, IL-13, etc.) and IL-10 may modulate Th1 proinflammatory responses (18, 19). In scrub typhus patients, dominant Th1 immune responses have been observed, as evidenced by highly increased IFN-γ, TNF-α and CXCL10, while levels of Th2-associated chemokines were undetectable or marginally induced (20, 21). Blood brain barrier (BBB) leakage, as measured by significantly higher albumin index in cerebrospinal fluid has been confirmed in scrub typhus patients (22). Moreover, the increased albumin index was positively correlated with the expression levels of an astrocyte marker (glial fibrillary acidic protein), indicating the activation of astrocytes in the brain of scrub typhus patients (22). In a rhesus macaque (*Macaca mulatta*) model of *Ot* infection, a preferential development of a Th1 immune response was also observed as characterized by upregulation of serum IFN-γ as well as other inflammatory cytokines including TNF-α, IL-15, IL-6, IL-18, regulatory IL-1ra, IL-8, and G-CSF (23). However, in-depth investigation of *Ot*-related neuroinflammation and BBB damage in humans or non-human primate models are lacking.

Using lethal mouse models of scrub typhus induced by *Ot* Karp strain, our group has demonstrated the type 1-skewed neuroinflammation, cellular activation and vascular activation/damage in the brain, even though the brain tissues contain relatively low bacterial burdens compared with other organs such as lungs (24, 25). In mouse cortex and cerebellum sections, there are increased infiltration of immune cells including T cells and macrophages, as well as the activated brain resident microglia and astrocytes at the peak of infection (days 6-10) (24), which correlated with endothelial damage as judged by increased ICAM1-positive staining in *Ot*-infected brain tissues (4, 24). In addition to *Ot* infection, animal studies showed that other Rickettsial infections also induce neurological disorder. In a *Rickettsia typhi* study, infected RAG1^-/-^ mice exhibited lethal neurological disorders with a massive expansion of microglia and neuronal cell death in a persistent infection stage (26). BBB damage and intracerebral microhemorrhages were also reported in a mouse model of *R. australis* infection (27). However, the mechanism of CNS infection and AES in scrub typhus is unknown, mainly due to the lack of appropriate animal models and the requirement of biosafety level 3 practices and containment.

In the current study, we wanted to further explore molecular mechanisms underlying neurological pathogenesis in severe scrub typhus via utilizing a lethal C57BL/6 mouse model. To obtain an unbiased view of brain responses to *Ot* infection, we used comprehensive approaches, including brain tissue RNA-seq assay and qRT-PCR analyses of relevant genes, as well as *in vitro* infection of human and mouse microglial cell lines for assessing bacterial growth and inflammatory responses. This study provides the first line of evidence for microglial activation and selective activation of the IFN pathways, which may lead to excessive neuroinflammation and BBB disruption at severe stages of *Ot* infection.

## Materials and methods

### Mouse infection and ethics statement

Female C57BL/6 mice were purchased from Jackson Laboratory (stock #000664). Mice were maintained under specific pathogen-free conditions and used at 6-9 weeks of age, following protocols approved by the Institutional Animal Care and Use Committee (protocol # 1902006) at the University of Texas Medical Branch (UTMB) in Galveston, TX. All mouse infection studies were performed in the ABSL3 facility in the Galveston National Laboratory located at UTMB; all tissue processing and analysis procedures were performed in the BSL3 or BSL2 facilities. UTMB operates to comply with the USDA Animal Welfare Act (Public Law 89–544), the Health Research Extension Act of 1985 (Public Law 99–158), the Public Health Service Policy on Humane Care and Use of Laboratory Animals, and the NAS Guide for the Care and Use of Laboratory Animals (ISBN-13). UTMB is a registered Research Facility under the Animal Welfare Act and has a current assurance on file with the Office of Laboratory Animal Welfare, in compliance with NIH Policy. Mice (5/group) were inoculated intravenously (i.v.) with 6 × 10^4^ FFU (200 µl) of *Ot* Karp strain or PBS (day 0) and monitored daily for weight loss, signs of disease, and survival. Animals were euthanized at day 0, 2, 6 and 10 post infection for tissue collection (28–30). Brain tissues were stored in *RNAlater* (Qiagen) and incubated in 4°C overnight for inactivation of bacteria. This animal experiment has been conducted independently three times.

### Cell culture and bacterial stock preparation

Vero cells (African green monkey kidney epithelial cells) were cultured in MEM medium (Gibco) containing 10% fetal bovine serum (FBS), 100 U/mL penicillin, and 100 µg/mL streptomycin. Bacterial stock was prepared, as in our previous reports (29, 30). BV2 cells were cultured with DMEM medium plus 10% FBS; C20 was cultured in DMEM/F12 medium (Gibco) supplemented with 1% FBS and 1×N-2 supplement (Gibco) (31). Cells were infected with bacteria (MOI 10) and harvested at 24 and 72 hours (h). Uninfected cells were used as negative controls.

### Quantitative reverse transcriptase-PCR

Tissues were homogenized using metal beads in a BeadBlaster 24 Microtube Homogenizer (Benchmark Scientific) with an RLT lysis buffer (Qiagen). Cultured cells were also harvested and lysed by using RTL lysis buffer. RNA was extracted by using RNeasy Mini kits (Qiagen) and used for cDNA synthesis with an iScript Reverse Transcription kit (Bio-Rad). cDNA was amplified in a 10 μL reaction mixture containing 5 μL of iTaq SYBR Green Supermix (Bio-Rad) and 5 μM each of gene-specific forward and reverse primers. The PCR assays were denatured for 30s at 95°C, followed by 40 cycles of 15s at 95°C, and 60s at 60°C, by utilizing the CFX96 Touch real-time PCR detection system (Bio-Rad). Relative quantitation of mRNA expression was calculated using the 2^−ΔΔCt^ method. The primers are listed in **Supplementary Table 1**.

### IF staining and fluorescent microscopy

Brain tissues were fixed in 4% paraformaldehyde (PFA) and embedded in Tissue-Tek OCT, as described in our previous report (24). Briefly, cryosections (6 µm) were fixed for 10 min in ice-cold acetone and rehydrated with PBS. For immunofluorescent staining, sections were permeabilized and blocked using 5% normal goat serum, 0.2% Triton X-100 in PBS for 1 h, followed by primary antibody incubation overnight at 4°C. The anti-IBA1 Ab (ab178846) was purchased from Abcam and Biolegend, respectively. The Alexa Fluor555-conjugated or Alexa Fluor488-conjugated anti-rabbit IgG (H+L), F(ab’)2 Fragment secondary antibodies (Cell Signaling Technology, 1:500 dilution) were used to stain the sections for 1 h at room temperature, followed by three washes with PBS. The TrueVIEW Autofluorescence Quenching Kit (Vector Laboratories) was applied to diminish unwanted autofluorescence and the microscope slides were covered by using Fluoroshield Mounting Medium with DAPI (Abcam). Images were captured using a Carl Zeiss Axio Observer fluorescence microscope or a confocal microscope Zeiss LSM 880 with Airyscan. For each mouse, at least three fixed-frozen sections were included for each experiment. All images were captured with the same conditions of laser power and detector gain.

### RNA-seq assay and data analysis

Brain RNA was extracted by using RNeasy RNA Isolation kit (Qiagen). Samples (3 mice/group) were sent to LC Sciences (Houston, TX) for RNA purity/quantity assessment via Bioanalyzer 2100 and RNA 6000 Nano LabChip Kit (Agilent), mRNA extraction, cDNA libraries construction, and sequencing by using the Illumina Novaseq™ 6000. The Mus musculus genome build mm10 was used as a reference genome. Quality control analysis was done by using FastQC (32) and RSeQC6 (33). Semi-supervised hierarchical clustering analysis and heatmap generation were performed by using the Morpheus tool (https://software.broadinstitute.org/morpheus). Principal component analysis and volcano plot were done in R using *PCAtools* (34) and *EnhancedVolcano* (35) packages. The RNA-seq data presented herein were deposited in NCBI’s Gene Expression Omnibus, accessible through GEO Series accession number GSE227525 (https://www.ncbi.nlm.nih.gov/geo/query/acc.cgi?acc=GSE227525).

### Quantitative PCR for measuring bacterial burdens

To determine bacterial burdens, DNA was extracted from brain tissues via a DNeasy Blood & Tissue Kit (Qiagen) and used for qPCR assays, as previously described (28, 36, 37). The primers were OtsuF630 (5’-AACTGATTTTATTCAAACTAATGCTGCT-3’) and OtsuR747 (5’-TATGCCTGAGTAAGATACGTGAATGGAATT-3’) (Integrated DNA Technologies). Bacterial loads were normalized to total nanogram (ng) of DNA per µL for the same sample, and data are expressed as the gene copy number of 47-kDa protein per ng of DNA. The copy number for the 47-kDa gene was determined by known concentrations of a control plasmid containing single-copy insert of the gene. Gene copy numbers were determined via serial dilutions (10-fold) of the *Ot* Karp 47-kDa plasmid.

### GSEA analysis

Pathway enrichment analysis using gene set enrichment analysis with GSEA version 3.0 was performed with a ranked gene list (38, 39). Specifically, ranked gene list is prepared from the differential gene expression analysis by using the following metrics: sign of the Log_2_fold change multiplied by the inverse of the p-value. *GSEAPreranked* was run with 2000 permutations. Primary gene sets investigated were obtained from the MSigDB and published reports (40, 41). GSEA FDR Q <0.05 cutoff was applied to examine enriched gene sets in our dataset.

### Cytoscape analysis

Enrichment results from the GSEA analysis were visualized using Cytoscape (42). GSEA output files were uploaded in the EnrichmentMap app of Cytoscape and FDR Q value cut off was set to 0.01. Clusters are automatically defined using AutoAnnotate Cytoscape app.

### Statistical analysis

Data were presented as mean ± standard error of mean (SEM). Differences between individual treatment and control groups were determined by using unpaired Student’s t test, utilizing Welch’s correction when appropriate. One-way ANOVA was used for multiple group comparisons, with a Tukey’s *Post Hoc* for comparisons between groups. Two-way ANOVA was used for the statistical analysis of body weight change. Log-rank (Mantel-Cox) test was used for survival curve analysis. All data were analyzed by using GraphPad Prism software 8.0. Statistically significant values are denoted as *, *p* < 0.05; **; *p* < 0.01, ***; *p* < 0.001, and ****; *p* < 0.0001, respectively. All *in vitro* cell culture experiments were performed three times independently, with triplicate samples obtained each time. All qRT-PCR and qPCR assays (4-5 samples/group) were repeated twice independently.

## Results

### Severe *Ot* infection leads to widespread changes in gene expression in the brain

**Figure 1:**
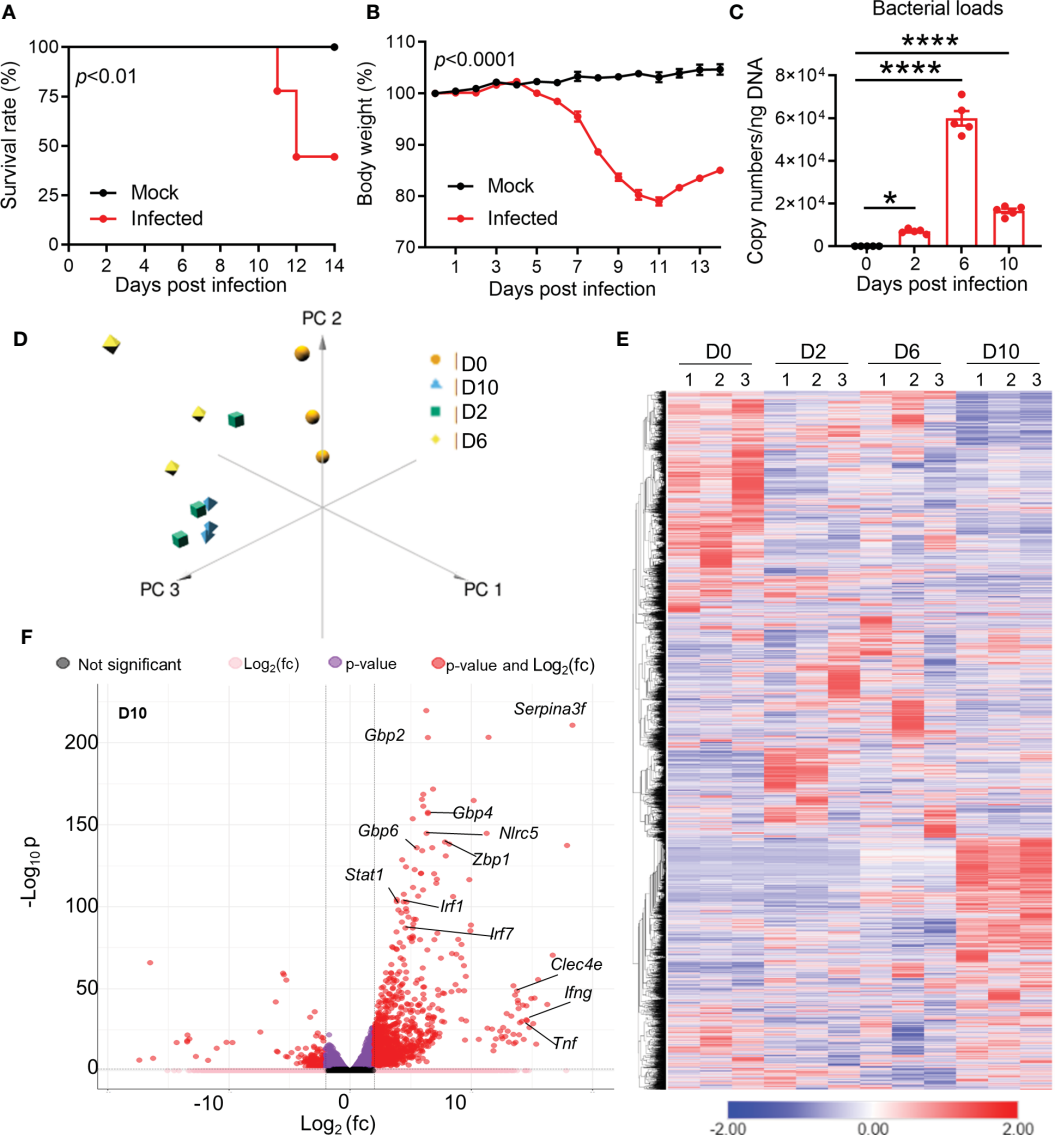
Dynamic brain transcriptomes of mice following *Ot* infection. B6 mice (5/group) were injected i.v. with *Ot* (6 × 10^4^ FFU) or PBS (used as a mock or D0 controls). Brain cortexes were harvested at days 2, 6, and 10 post-infections, followed by RNA isolation. **(A)** Animal survival rates. **(B)** Body weight changes. **(C)** Bacterial burdens in the brain tissues. This animal experiment has been conducted independently three times and the data are shown from a single representative experiment. ****, p < 0.0001. **(D)** Principal component analysis of RNA-seq data of mouse brain at days 2, 6, and 10. Three samples in each group were performed for RNA-seq analysis. **(E)** Unsupervised clustering of brain transcriptomes. **(F)** Volcano plot analysis of differentially expressed genes at day 10 vs day 0.

To understand the brain pathogenesis in severe *Ot* infection, we intravenously infected mice with lethal dose of bacteria (6 × 10^4^ FFU) and collected brain tissues at days 2 (incubation period), 6 (disease onset), and 10 (disease peak), respectively. As in our previous reports (28, 37), infected mice started body weight loss around day 7, and approximately half of mice expired between days 11 and 14 (**Figures 1A, B**). Brain bacterial burdens were detectable at day 2, reached the peak at day 6, and reduced at day 10 (**Figure 1C**). We used RNA-seq based transcriptomics profiling of brain cortex at the indicated timepoints (**Figure 1D**). Mice with PBS injection were used as mock controls (day 0). We used Principal Component Analysis (PCA) and semi-supervised Hierarchical Clustering Analysis (HCA) to understand the pattern of gene expression changes in the brain during the infection. PCA analysis revealed a clear separation between infected samples compared to uninfected brain tissues (**Figure 1D**). Infected samples from the same timepoints were closer to each other as compared to others, suggesting a consistent difference in the brain transcriptomes at different stages of infection. HCA analysis also followed the same trends, with clear differences in gene expression at different timepoints (**Figure 1E**). Day 2 samples had a total of 1,333 differentially expressed genes, while days 6 and 10 samples contacted a total of 1,478 and 2,469 differentially expressed genes, respectively. Several genes previously reported to be involved in host responses against *Ot* (24, 43) were differentially expressed in infected brains, including interferon-stimulated genes (ISGs) that are critical components of immune response to microbial infections (*Gbp2/4/6*, *Stat1*, *Mx1* and *Irf7)* at day 10 (**Figure 1F**). Consistent with our previous reports (24, 29), *IFNg* and *Tnf* were the dominate type-1 immune markers in infected brains. Together, the RNA-seq transcriptomics revealed widespread dysregulation of gene expression with strong type 1 immunity in *Ot*-infected brain tissues.

### *Ot* infection results in the activation of IFN signaling pathways and neuroinflammation

**Figure 2:**
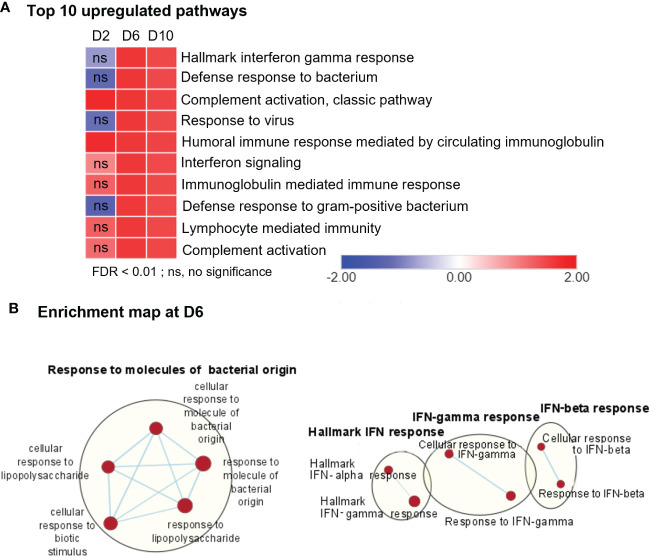
RNA-seq analyses of differentially expressed genes, showing upregulation in immune signaling in *Ot*-infected mouse brains. Brain RNA-seq experiment was performed, as in **Figure 1**. **(A)** Pathway enrichment analysis of differential expressed genes by using GSEA shows top 10 upregulated pathways in infected brain samples as compared to mocks. **(B)** Cytoscape enrichment map (FDR Q value < 0.01) of GSEA pathways enriched in upregulated genes in infected mouse brain at day 6 compared to mocks. Clusters of nodes were labeled using Auto Annotate feature of Cytoscape application. Red nodes represent upregulated gene set enrichment, while blue represents downregulated gene sets. ns, not significant.

To define cellular processes that may cause neuropathology in *Ot*-infected brains during disease progression, we performed comparative pathway analyses on the transcriptomics data from brains (mocks versus days 2, 6, and 10, respectively) by using the Gene Set Enrichment Analysis (GSEA) software, as well as the Cytoscape Enrichment Map to visualize the biological processes that may play a role in host response against bacterial infection. Consistent with our previous PCR- and NanoString-based studies (4, 24, 29), day 2-vs-mock GSEA analysis of RNA-seq data revealed minor, but statistically significant, upregulation in a few biological pathways, including “complement activation pathway” and “humoral immune response mediated by circulating immunoglobulin” (**Figure 2A**, **Supplementary Figure 1A**). In contrast, we observed strong enrichment of several pathways related to immune signaling and inflammation for day 6- and day 10-versus-mock comparison. The notable top 10 pathways were related to “Hallmark interferon gamma response,” “defense response to bacteria,” and “Immunoglobulin mediated immunity” (**Figures 2A, B**, **Supplementary Figure 1B**). To further validate genes differentially induced at days 6 and 10, we analyzed their transcriptomic profiles by using mSigDB Hallmark gene data set (**Supplementary Figure 2**). Consistently, “Hallmark interferon gamma response,” “Hallmark interferon alpha response,” “IL-6, JAK-STAT signaling,” and “TNF signaling via NF-κB” were among the top 10 pathways induced at days 6 and 10, respectively, as compared to control brains. The lack of an enrichment for these pathways at day 2, suggested to us that immune signaling in response to *Ot* infection peaked around day 6 and had sustained activation at day 10 (prior to host death).

### *Ot* infection alters the expression of blood-brain barrier genes

**Figure 3:**
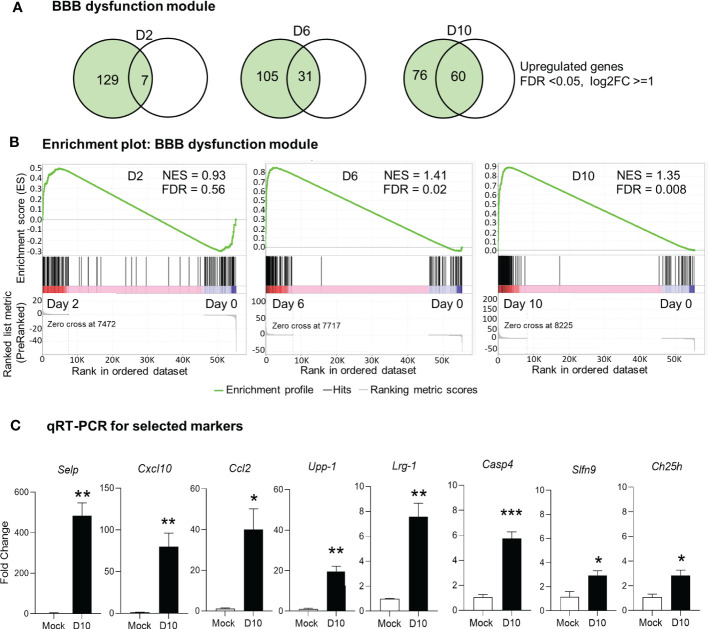
Alteration of blood brain barrier in *Ot*-infected mice. Brain RNA-seq experiment was performed, as in **Figure 1**. **(A)** Venn diagram shows the numbers of unique and shared differentially expressed genes for each comparison. **(B)** GSEA analysis identifies significant enrichment of blood brain barrier dysfunction module at days 6 and10. **(C)** qRT-PCR assay confirms the upregulation of blood brain barrier related genes at day 10 as compared to mocks. A two-tailed student t test was used for comparison between two groups. *, *p* < 0.05; **, *p* < 0.01; ***, p < 0.001. The qRT-PCR data (4-5 samples/group) were from one experiment representative of three performed independently.

It is well known that BBB tightly regulates the movement of biological substances between blood and neural cells and is required for maintaining brain homeostasis. BBB disruption is often observed in various pathological conditions, including severe bacterial infection (4, 22, 44). Our previous histological analyses of *Ot-*infected mouse brains have revealed prominent endothelial cell damage, with increased recruitment of CD3^+^ T cells and CD45^+^ leukocytes to the cortex and cerebellum (24, 30). Other groups have recently reported a core of BBB genes that can be used as the signature biomarkers of BBB disruption in various neurological diseases (40), as this core module includes 136 genes critical for various BBB functions (**Supplementary Table 2**). To assess if BBB dysfunction contributed to our observed mouse mortality, we considered and examined these BBB signature genes in our analyses of transcriptomics. Strikingly, we found that many upregulated genes (FDR <0.05, log2FC = 1) in our dataset were consistent and overlapped with the core BBB signature. For day 2 samples, this overlap was minimal and only 7 upregulated genes were found (**Figures 3A, B**), which was consistent with our previous report of minimal vascular damage at initial infection (24). As expected, a considerable overlap was found between our dataset and the reported BBB dysfunction module (40), with 31 matched genes at day 6, and 60 genes at day 10, respectively (**Figures 3A, B**). GSEA analysis also showed an enrichment of BBB dysfunction module at days 6 and 10 (**Figure 3B**). We further analyzed several BBB genes at day 10 via qRT-PCR and confirmed a significant increase in *Cxcl10*, *Casp4*, *Upp1*, *Ch25h*, *Selp*, *Lrg1*, *Ccl2*, and *Slfn9* expression as compared to mocks (day 0) (**Figure 3C**). Together, these data indicate a disruption in BBB during severe stages of lethal *Ot* infection.

### Transcriptional regulation by STAT1/STATdrives type responses against *Ot* infection in mice

**Figure 4:**
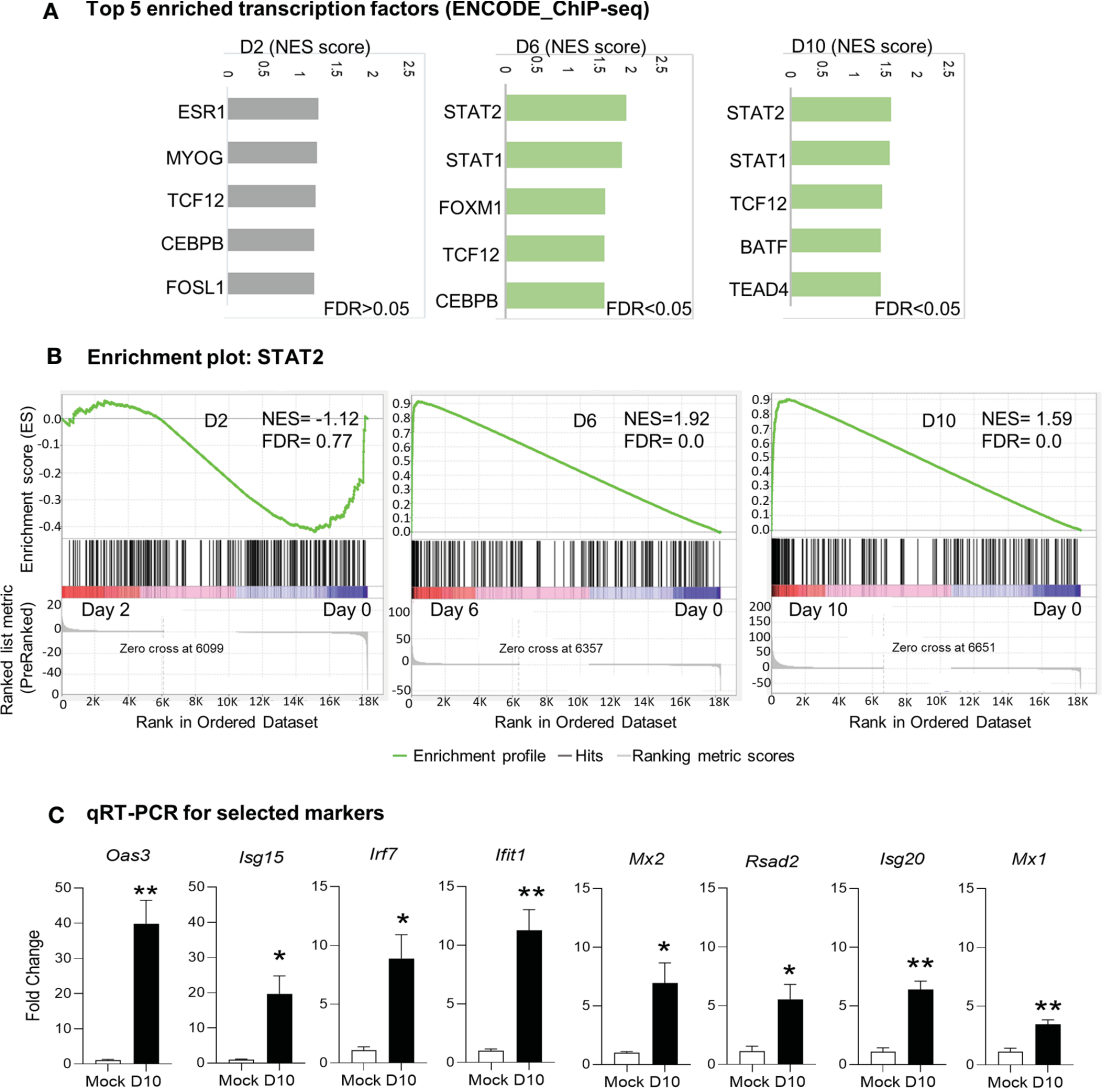
Identification of putative transcription factors responsible for differential gene expression during *Ot* infection. Brain RNA-seq experiment was performed as in **Figure 1**. **(A)** GSEA analysis to identify top 5 enriched transcription factors in mouse brain. **(B)** Enrichment plots for the transcription factor STAT2. **(C)** qRT-PCR assay confirms the upregulation of interferon-stimulated genes at day 10 as compared to mocks. A two-tailed student t test was used for comparison between two groups. *, *p* < 0.05; **, *p* < 0.01. The qRT-PCR experiments were performed twice independently with 4-5 samples per group, and the data are shown from a single representative experiment.

Since we observed activation of immune signaling in the brain as the main biological process in response to *Ot* infection, we next examined key transcription factors that might drive this host signaling response. We performed GSEA analysis by using ENCODE ChIP-Seq dataset. The significant enrichment of transcription factors critical for immune signaling was evident at days 6 and 10 (FDR <0.05), but not detectable at day 2 (FDR >0.05; **Figure 4**). The top enriched 5 transcription factors at day 6 were STAT2, STAT1, FOXM1, TCF12, and CEBPB; at day 10, STAT2, STAT1, TCF12, BATF and TEAD4 were identified as top 5 enriched transcription factors (**Figures 4A, B**). Our qRT-PCR data further confirmed RNA-seq findings, judged by significantly increased transcript levels of *Mx1*, *Mx2*, *Isg20*, *Isg15*, *Irf7*, *Oas3*, *Ifit1*, and *Rsad2* at day 10 as compared to mocks (day 0) (**Figure 4C**). Importantly, STAT1 and STAT2 were both identified as top enriched transcription factors at days 6 and 10 data sets, suggesting a critical role for IFN signaling in mediating neuroinflammation during *Ot* infection.

### Reactive microglia state is induced in response to *Ot* infection

**Figure 5:**
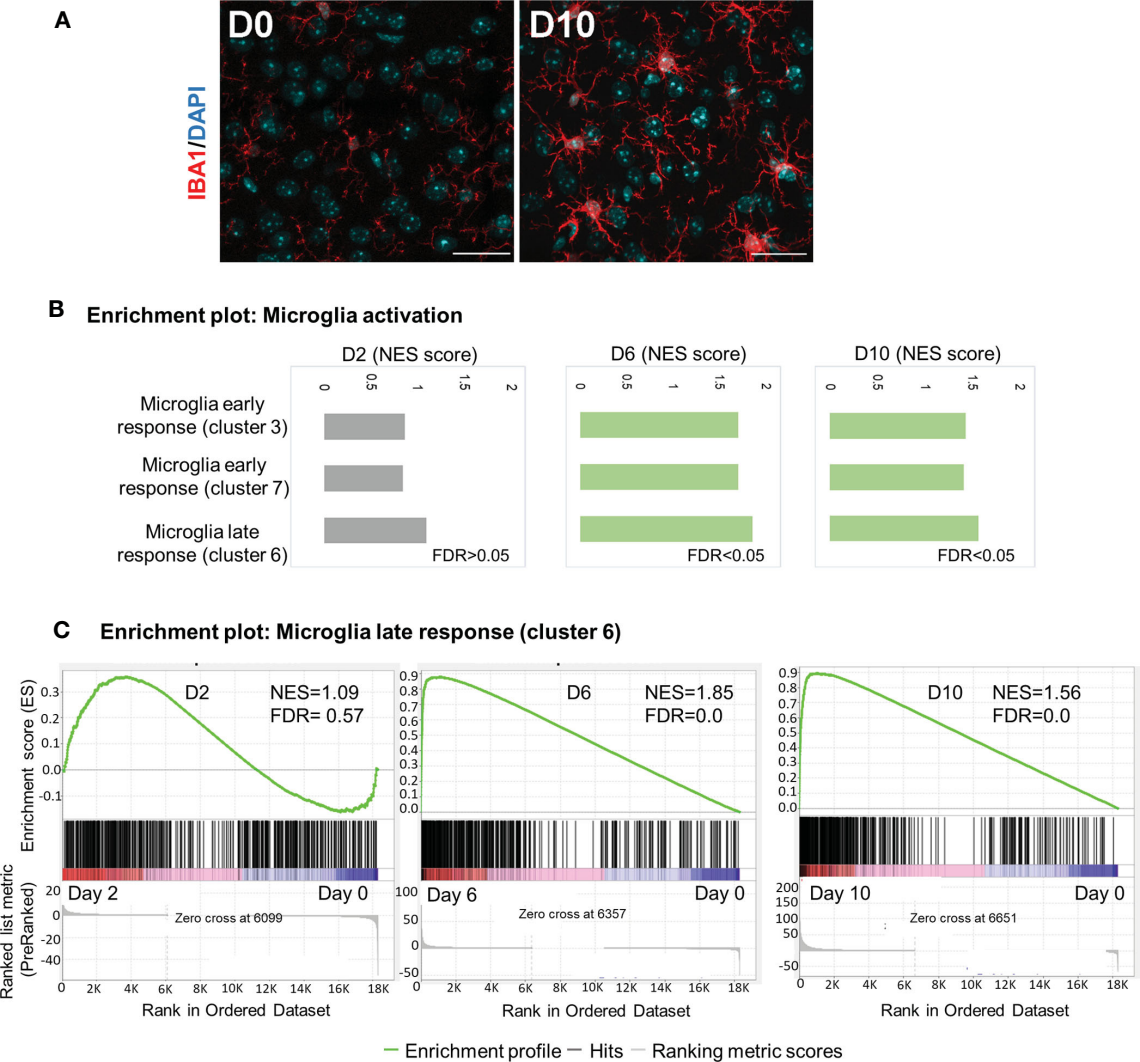
Activation of microglia in mouse brain in response to *Ot* infection. Brain RNA-seq experiment was performed as in **Figure 1**. **(A)** Activation of microglia at day 0 and 10 was evaluated by immunostaining (IBA1: red; DAPI: blue; Scale bars = 50 μm). Images were shown from one experiment representative of three performed independently (5 mice/group). **(B)** GSEA analysis identifies activation of ‘early response’ and ‘late response’ microglia gene expression signatures. **(C)** Enrichment plots for microglia ‘late response’ gene expression module at days 2, 6 and 10.

Microglia are brain-resident myeloid cells that are considered immune sentinels, playing key roles in brain homoeostasis during physiological and pathological conditions. To determine microglia activation following *Ot* infection, we stained mouse brain sections with IBA1 (a microglia marker). While the control samples contained resting microglia, characterized by long, ramified processes with comparatively small cell bodies, microglia activation was significant at day 10, as judged by their increased branching and lengthening of processes and enlarged cell bodies (**Figure 5A**). Mathys, et al. have recently used single-cell RNA-seq and identified a disease stage-specific microglia gene signature in a neurodegeneration model with Alzheimer’s disease-like phenotype, defining microglia reactive states as “early response” (a transient intermediate activation of microglia that proliferate) and “late response” (reactive microglia), respectively (41). To assess microglia activation state during *Ot* infection, we utilized Mathy’s reported analysis strategy. Interestingly, we found a significant enrichment of gene sets for early response (cluster 3/cluster7) and late response (cluster 6) at days 6 and 10, as compared to control brains by using GSEA (**Figures 5B, C**). Such trends were consistent with increased number of IBA1^+^ cells in infected brain tissues (**Figure 5A**). Notably, late-response-gene set had higher NES compared to early-response-gene set, suggesting a dominant response from reactive microglia during *Ot* infection (**Figure 5**). Together, our data supports a role of microglia activation in driving neuroinflammation in the brain of *Ot-*infected mice.

Astrocytes, also known as astroglia, are characteristic star-shaped glial cells in the brain and are often thought of as “nurse” cells for neurons in the brain parenchyma. Reactive astrocytes in CNS injury or disease can be polarized to A1 and A2 types that are neurotoxic and neuroprotective, respectively (45, 46). Since A1 astrocytes are induced by classically activated neuroinflammatory microglia (45), we speculated that severe *Ot* infection may also promote A1-like responses in the brain. We then analyzed our RNA-seq data for gene profiles associated with astrocyte activation and found that most pan-astrocytic markers were upregulated at day 10, as compared to those of day 0. Importantly, most A1-specific genes were also upregulated, while 70% of A2-specific markers were not altered or slightly decreased at the severe stage of infection (**Supplementary Figure 3**). Oure data suggest a strong brain A1-like responses in severe *Ot* infection, and these neurotoxic astrocytes may contribute to *Ot*-induced neuroinflammation and acute encephalitis.

**Figure 6:**
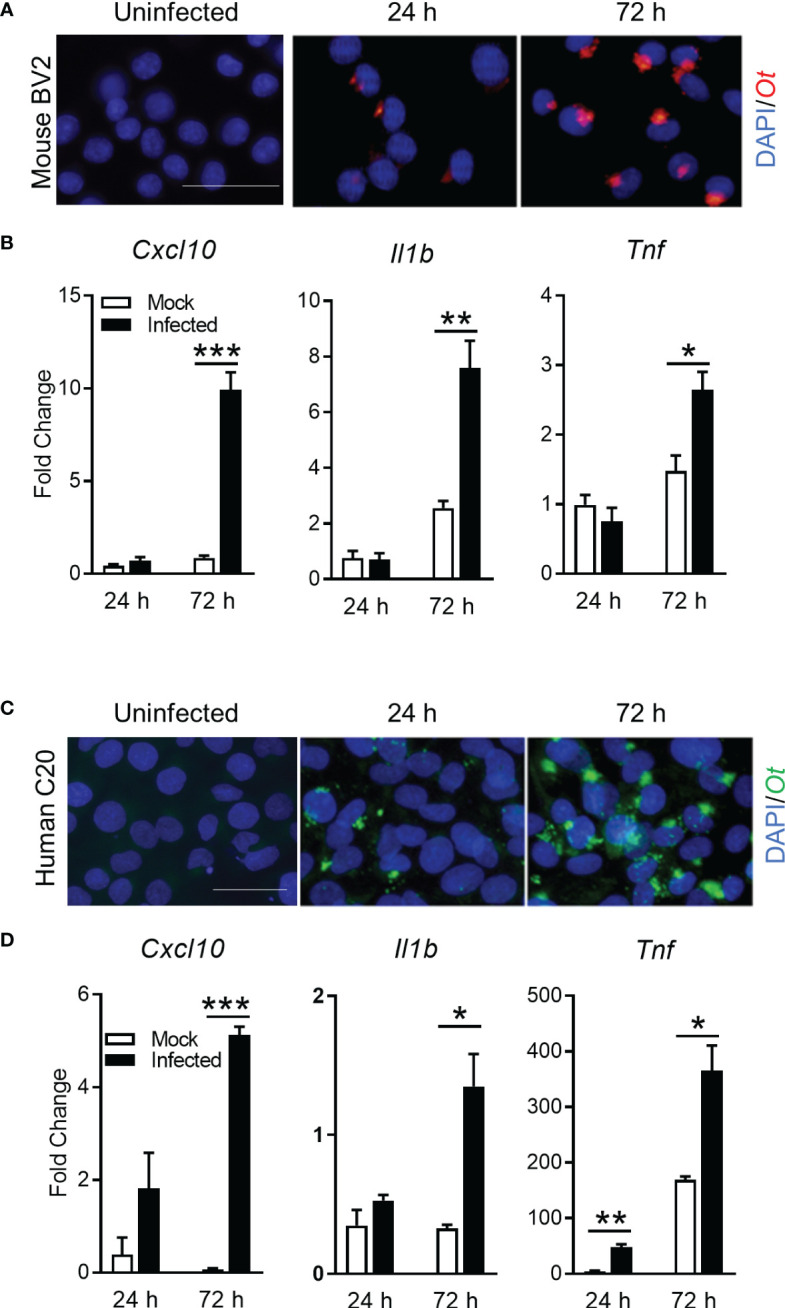
*Ot* bacteria replicate in microglial cell lines and induce inflammatory responses. Mouse BV2 and human C20 microglial cell lines were infected with *Ot* (MOI 10) *in vitro*. Uninfected cells were used as controls. Cells were harvested at 24 and 72 h. **(A, C)** Bacteria were detected by immunostaining using anti-TSA56 antibody. Scale bars = 50 μm. **(B, D)** Transcriptive levels of inflammatory gene *Cxcl10*, *IL1b*, and *Tnf* were analyzed by qRT-PCR. A two-tailed student t test was used for comparison between two groups. *, *p* < 0.05; **, *p* < 0.01; ***, *p* < 0.001. These experiments were repeated twice independently with triplicate samples each time and the data were shown from a single representative experiment.

To support our conclusion, we further investigated whether *Ot* replicated in microglia and promoted inflammation *in vitro*. We infected mouse and human microglia cell lines (BV2 and C20, respectively) with bacteria (MOI 10). Immunofluorescence staining clearly indicated established *Ot* infection in mouse microglia (**Figure 6A**). Furthermore, qRT-PCR analysis of proinflammatory genes indicated significantly increased levels of *Cxcl10*, *Il-1b*, and *Tnf*, especially at 72 h post-infection (**Figure 6B**). Similar results were also found in human microglia C20 (**Figures 6C, D**). The time-dependent increase in bacterial burdens of C20 cells confirmed productive intracellular replication (**Supplementary Figure 4**). Together, our *in vitro* data demonstrate *Ot*-induced bacterial growth and a pro-inflammatory response in microglia, potentially leading to neuroinflammation and subsequent brain damage.

## Discussion

Neurological manifestations and sequelae of scrub typhus have long been overlooked. Antibiotics for the treatment of scrub typhus are not routinely advised for empirical treatment of CNS infections, leading to chronic sequelae of infection (14). Although clinical reports imply that *Ot* infection is the significant cause of AES in the endemic region, there is limited mechanistic research of *Ot*-induced AES mainly due to the shortage of appropriate animal models. To better understand the mechanism of CNS disorder in scrub typhus, we performed a kinetic study by using our established mouse model (24, 30), analyzed brain tissue samples by RNA-seq and immunostaining, and conducted *in vitro* infection studies with microglial cell cultures. This study provided several lines of new evidence for IFN responses, neuroinflammation, microglial activation, and BBB dysregulation during severe scrub typhus in mice.

Firstly, our data revealed the significant upregulation of IFN signaling in the brains of severe *Ot* infection. The significant IFN-I upregulation in *Ot* infection was not observed at day 2, but at days 6 and 10, respectively (**Figure 1**, **Supplementary Figure 1**). This result was consistent with a report by Min et. al., who showed the increased IFNβ proteins in mouse plasma at day 12 of *Ot* infection (47). High level of IFN-I may help control *Ot* infection in the brains and other organs, as we found significantly decreased bacterial burdens at day 10 compared to those at day 6 (**Figure 1C**). However, IFN-I may also contribute to neurological diseases, due to its neurotoxic effect on brain cells (48), and IFN-I induced by systemic infection can also hinder CNS vascular repair following brain injury (49). While our RNA-seq data were consistent with those reports, revealing the disrupted BBB at day 10 (**Figure 3**), further investigation is necessary to elucidate the essential function of IFN-I in *Ot*-induced acute and persistent neurological disorders.

Obligate bacterial pathogens that inhabit the host cytosol are often resistant to IFN-I, but such bacteria are sensitive to control mechanisms mediated by IFN-γ (50). A reported *in vitro* study with *Ot* Gilliam strain and BALB/3T3 mouse fibroblasts has indicated that IFN-γ significantly inhibits *Ot* growth, but also has toxic effects to the infected cells (51). In this study, the upregulation of cerebral IFN-γ expression at days 6 and 10 may be attributed to cellular recruitment to the CNS, including Th1 and cytotoxic T cells, as observed in our previous immunostaining study (24). These infiltrates may contribute to the activation of resident glial cells and aid in the clearance of bacteria. Given that inducible nitric oxide synthase (iNOS) plays a crucial role in regulating *Ot* growth *in vitro* (25), it is plausible that IFN-γ may enhance iNOS signaling in microglia and infiltrating macrophages/monocytes, leading to bacterial elimination in the brain. Our RNA-seq data herein also showed the activation of complement in inflamed brains, consistent with previous findings from mouse blood samples (52). Furthermore, activated microglia are known to produce neurotoxic TNF-α, which may interact synergistically with IFN-γ to enhance inflammatory microglial responses (53). Such responses collectively can drive caspase-8/FADD-mediated PANoptosis, a process that can contribute to neurodegeneration (54). Therefore, the sustained cytokine/chemokine production may cause neuroinflammation, brain damage, and host death.

Secondly, our confocal microscopy study provided strong evidence for intense microglia activation at day 10 (**Figure 5**), even when brain bacteria were well under control (**Figure 1**). Our previously reported immunostaining study has revealed IBA1 and *Ot* colocalization in mouse brains (24). We noticed that IBA1-positive microglia at day 10 only exhibited a bushy morphology with enlarged cell bodies, but were not a final phagocytic amoeboid state, suggesting that they were highly activated at the severe disease stages (prior to host death). This was in sharp contrast to the relatively low brain bacterial burdens, implying a more important role of immunopathogenesis in brain injury. It was evident that *Ot* was capable of infecting and replicating within both human and mouse microglia *in vitro*, as indicated by the cytosolic growth of *Ot* observed through fluorescent staining at 72 h (**Figure 6**). Given our observation of high levels of IFN-γ in the brains of infected mice (**Figure 1**), as well as in our previous reports (4, 24), it is possible that microglia respond to IFN-γ stimulation and polarize to an M1-like phenotype (55), resulting in restricted *Ot* growth, as in our previous study on M1 macrophages (56). Consistent with a report of activated microglia in producing pro-inflammatory cytokines (57), we also found a significant increase in the expression of *Cxcl10, Il1b*, and *Tnf* (**Figure 6**). Among these cytokines, CXCL10 is a known typical type 1 immune chemokine, playing a key role for T cell recruitment and neuroinflammation in various infectious diseases, including COVID-19 (58–61).

It is known that IL-1β and TNF-α produced by activated microglia are detrimental during the brain infection, as they can cause neuronal death via direct effects on neurons or indirectly via glial production of neurotoxic substances (62, 63). Increased CNS IL-1β can further amplify microglial activation, leading to neuroinflammation that causes neuropathology. Furthermore, astrocytes can be activated to become either type 1 (A1) or type 2 (A2) cells, playing neurotoxic or neuroprotective roles in the CNS (45). Such astrocyte reactivation is known to be tightly regulated by activated microglia-derived inflammatory mediators, including TNF-α, IL-1α and C1q (45). As indicated in **Supplementary Figure 3**, *Ot* infection significantly upregulated A1-asscoated, but not A2-asscoated, genes. We speculated that activated microglia may also exacerbate inflammation and promote CNS damage via facilitating neurotoxic A1 polarization. Notably, TNF-α which is an indispensable mediator for microglia-induce A1 phenotype, were remarkably elevated in brain tissues (24) and *in vitro* microglial cultures by *Ot* infection (**Figure 6**). These results indicate that TNF-α might be the key inflammatory factor that mediates neuroinflammation in scrub typhus. However, systemic blockage of TNF signaling in mice at initial stage impairs anti-bacterial T cell responses (64), leading to unrestrained *Ot* replication and fatal outcomes (28). Further investigation is necessary to elucidate the temporal and tissue-specific functions of these inflammatory cytokines in scrub typhus.

Finally, our data collectively suggest the progression of BBB dysfunction during severe *Ot* infection. Clinical studies have reported signs of BBB damage in severe scrub typhus patients, as well as in other rickettsial spp. infection (22). *Ot* dissemination into the brain has been demonstrated in several mouse models (24, 25). However, clinical studies are limited, showing no specific biomarkers that could differentiate rickettsial from other neurological infections, such as tuberculosis meningitis and Japanese encephalitis virus (22). This study clearly revealed alterations of genes known to be associated with BBB disruption in different neurological diseases, but more importantly, a positive correlation of gene numbers and scrub typhus disease development (**Figure 3**). The core genes that were significantly increased at both days 6 and 10 (*Cxcl10*, *Casp4*, *Upp1*, *Ch25h*, *Selp*, *Lrg1*, *Ccl2*, *Slfn9*) were confirmed by qRT-PCR; they could serve as potential biomarkers of BBB disruption in *Ot* infection. Future molecular tests of specific biomarkers may help early detection of neurological damage and reduce the mortality of scrub typhus patients. It will also be important to determine the timing and underlying mechanisms of IFN responses in BBB disruption. Such studies will provide new insight into the prevention strategy of neurological disorder in scrub typhus.

**Figure 7:**
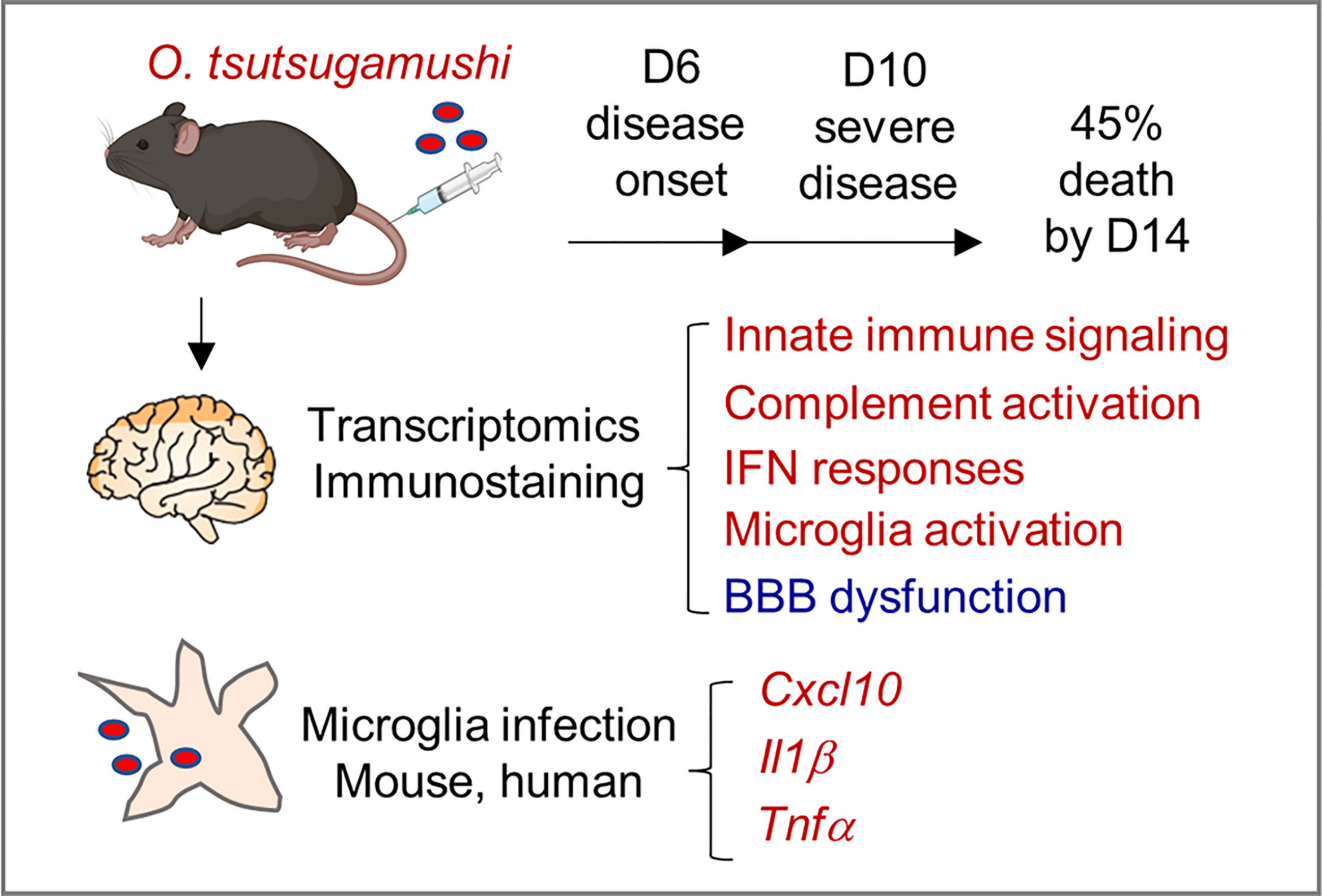
A schematic illustration of neuroinflammation in *Ot*-infected mice and microglia. Following i.v. infection with a lethal dose of *Ot* bacteria, B6 mice developed signs of disease (day 6), severe symptoms (day 10), with 45% mortality rates by day 14. Mouse brain transcriptomics at days 6 and 10 revealed the activation of innate immune signaling, complement activation, IFN responses, and brain-resident phagocyte microglia (in red), which correlated with reduction/dysregulation of the blood-brain barrier (in blue), particularly on day 10. Microglial cells were targets of *Ot* replication and cellular sources of proinflammatory gene expression (*Cxcl10*, *Il1b*, *Tnf*). These inflammatory responses collectively contributed to bacterial elimination in the brains, but also CNS damage in severe scrub typhus.

In summary, this study provided new lines of evidence for neuroinflammation and BBB dysregulation during severe scrub typhus in mice, as well as in *Ot*-infected microglial cell cultures (**Figure 7**). Mouse brain transcriptomics and immunostaining revealed highly activated innate immune signaling, complement activation, IFN responses, and the activation of brain-resident phagocyte microglia, which positively correlated with BBB dysregulation, particularly at late stages of scrub typhus. We clearly indicated that microglial cells were targets of *Ot* replication and cellular sources of proinflammatory gene expression (*Cxcl10, Il1b, Tnf*). We propose that these inflammatory responses collectively contribute to bacterial elimination in the brains, but also CNS damage in severe scrub typhus, and that immune-mediated tissue injury would sustain even when bacterial replication is under control. This study highlights the dominant type 1 immune responses, microglia activation, and BBB disruption in inflamed brain tissues, paving the way for future investigation of CNS dysregulation in scrub typhus and other rickettsial diseases. Further studies utilizing advanced technologies, such as single-cell RNA sequencing and other omics approaches, will provide a comprehensive understanding of the heterogeneous populations of brain resident cells and their precise functions in *Ot*-induced neurological disorders.

## Data availability statement

The datasets presented in this study can be found in online repositories. The names of the repository/repositories and accession number(s) can be found below: https://www.ncbi.nlm.nih.gov/geo/query/acc.cgi?acc=GSE227525.

## Ethics statement

The animal study was reviewed and approved by Mice were maintained under specific pathogen-free conditions and used at 6-9 weeks of age, following protocols approved by the Institutional Animal Care and Use Committee (protocol # 1902006) at the University of Texas Medical Branch (UTMB) in Galveston, TX.

## Author contributions

YL, LS, and PSa contributed to conception and design of the study. YL, HW and FO performed the experiments. YL, A, CG, LS and PSa organized the database and performed the statistical analysis. YL and A wrote the first draft of the manuscript. LS, PSu, PSa, YL and A revised the manuscript. All authors contributed to the article and approved the submitted version.

## Supplementary material

Supplementary Figure 1 Enrichment analysis of differentially expressed genes in response to *Ot* infection in mice. Brain RNA-seq experiment was performed as in Cytoscape enrichment map (FDR Q value < 0.01) of GSEA pathways enriched in upregulated genes in *Ot*-infected mouse brain tissue at days 2 **(A)** and 10 **(B)** post-infection compared to mocks.

Supplementary Figure 2 GSEA analysis using Hallmark gene sets of differentially expressed genes in *Ot*-infected mice. Brain RNA-seq experiment was performed, as in . GSEA analysis identifies hallmark gene sets at days 0, 6 and 10, respectively.

Supplementary Figure 3
*Ot* infection induces a type 1 astrocyte (A1) polarization in the brain. Brain RNA-seq experiment was performed, as in The FPKM fold changes of genes at day 10 were showed as a heat map with mock mice as controls. The selected genes were associated with pan reactive, A1- and A2-specific astrocytes. A two-tailed Student’s t test was used for statistical analysis of two groups. *, *p* < 0.05.

Supplementary Figure 4
*Ot* replication in a human C20 microglial cell line. C20 were infected with *Ot in vitro* (MOI 10) and uninfected cells were used as controls. Cells were harvested at indicated time points for bacterial burden measurement by qPCR. A one-way ANOVA with a Tukey’s multiple comparisons test was used for statistical analysis. This experiment was repeated twice independently with triplicate samples each time, and the data were shown from a single representative experiment.
