# Supplementary figures and images for: Brain transcriptomics reveal the activation of neuroinflammation pathways during acute Orientia tsutsugamushi infection in mice
Source: Front Immunol. 2023 Jun 22;14:1194881. doi: 10.3389/fimmu.2023.1194881 (PMC10326051; doi:10.3389/fimmu.2023.1194881)

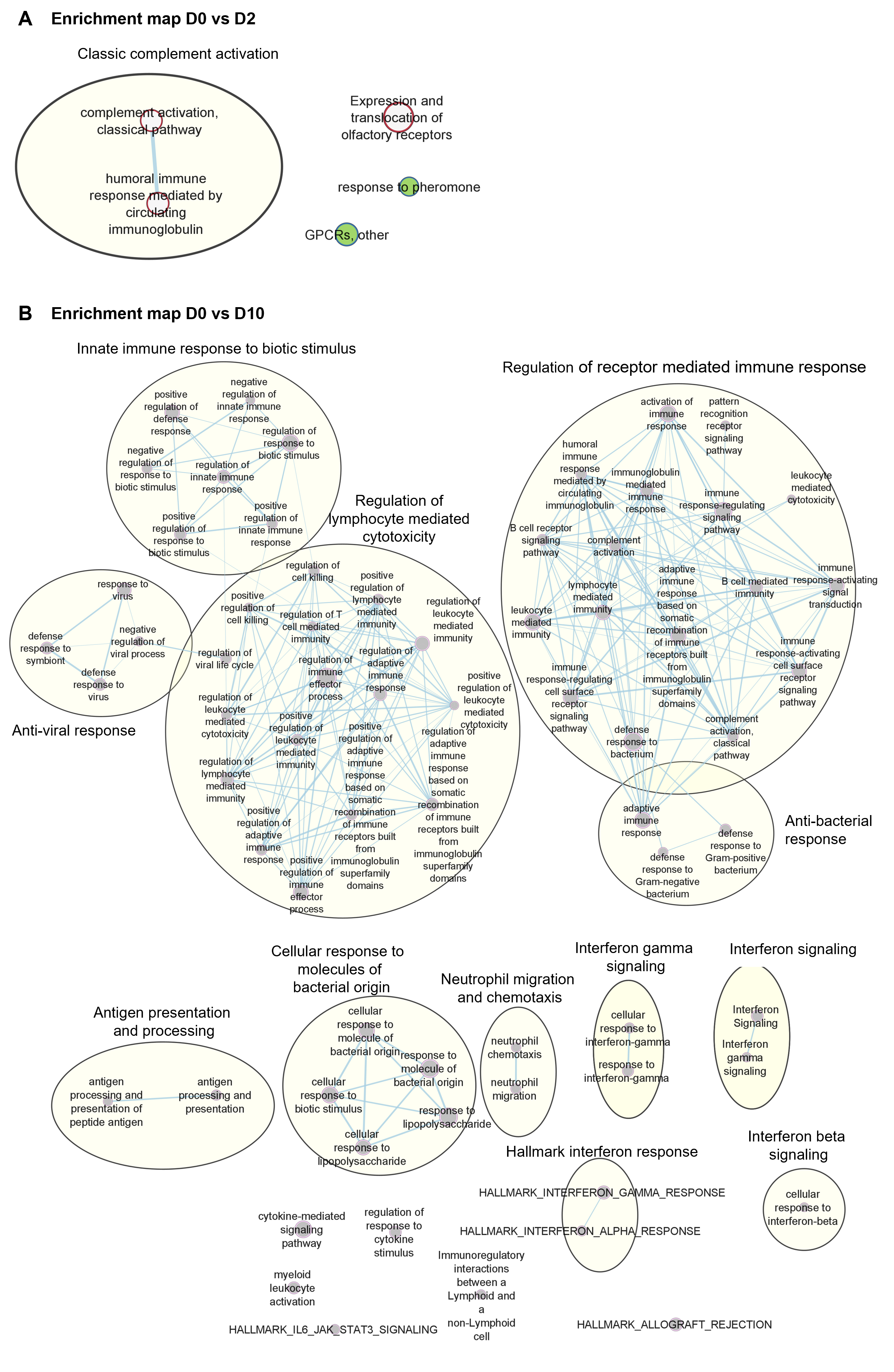

Supplement: Supplementary Figure 1 — Enrichment analysis of differentially expressed genes in response to Ot infection in mice. Brain RNA-seq experiment was performed as in Cytoscape enrichment map (FDR Q value < 0.01) of GSEA pathways enriched in upregulated genes in Ot-infected mouse brain tissue at days 2 (A) and 10 (B) post-infection compared to mocks. [file Image_1.tif]

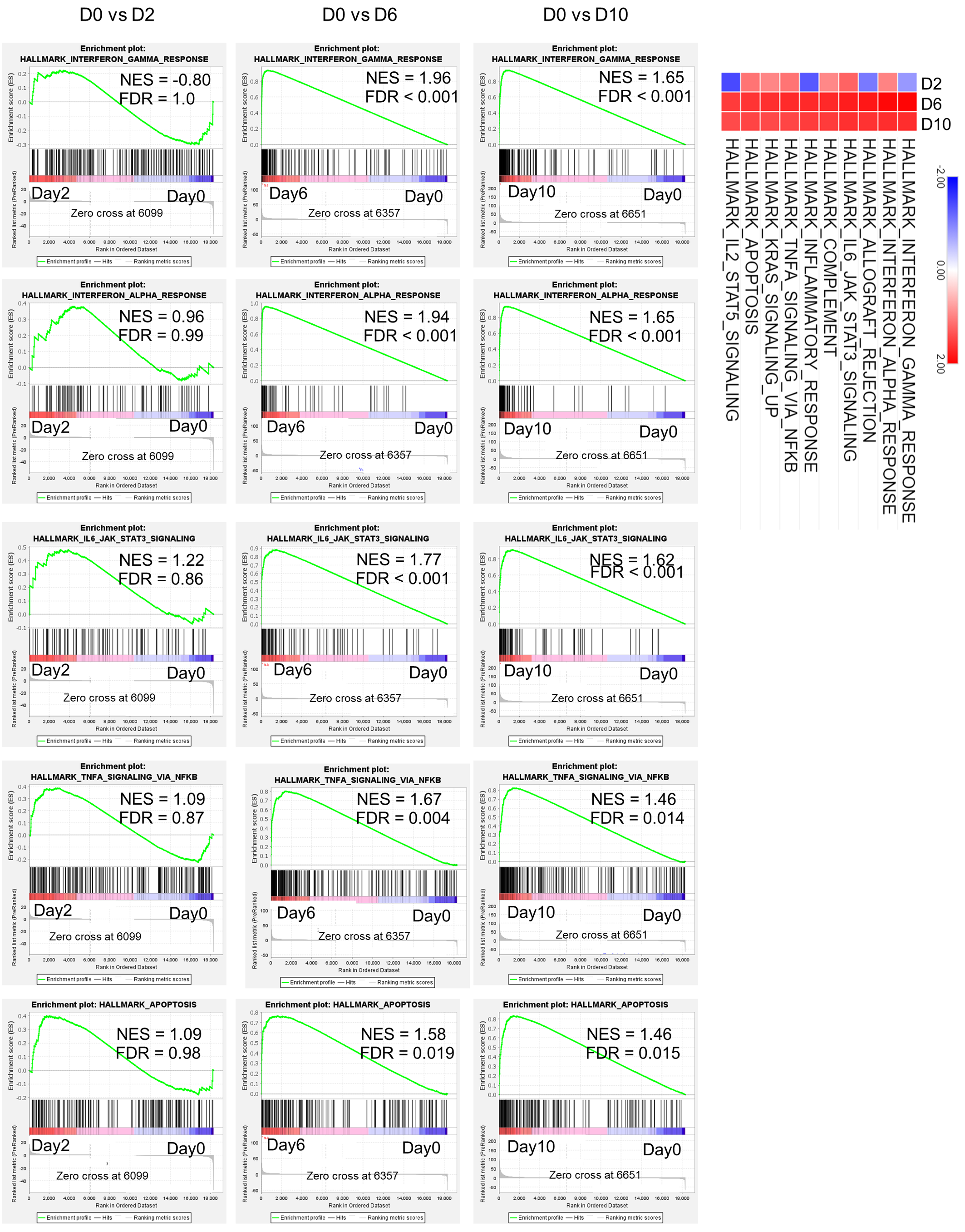

Supplement: Supplementary Figure 2 — GSEA analysis using Hallmark gene sets of differentially expressed genes in Ot-infected mice. Brain RNA-seq experiment was performed, as in . GSEA analysis identifies hallmark gene sets at days 0, 6 and 10, respectively. [file Image_2.tif]

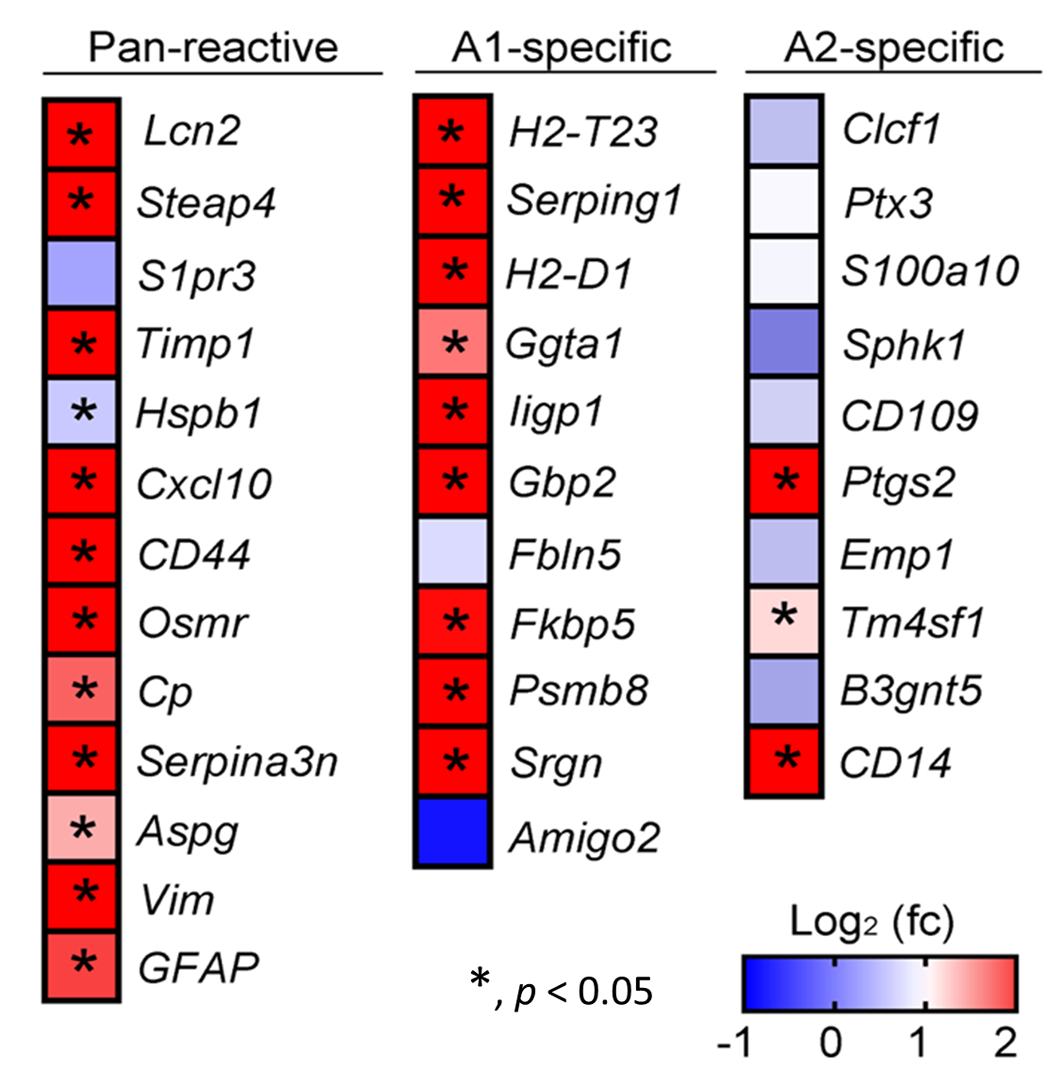

Supplement: Supplementary Figure 3 — Ot infection induces a type 1 astrocyte (A1) polarization in the brain. Brain RNA-seq experiment was performed, as in The FPKM fold changes of genes at day 10 were showed as a heat map with mock mice as controls. The selected genes were associated with pan reactive, A1- and A2-specific astrocytes. A two-tailed Student’s t test was used for statistical analysis of two groups. *, p < 0.05. [file Image_3.tif]

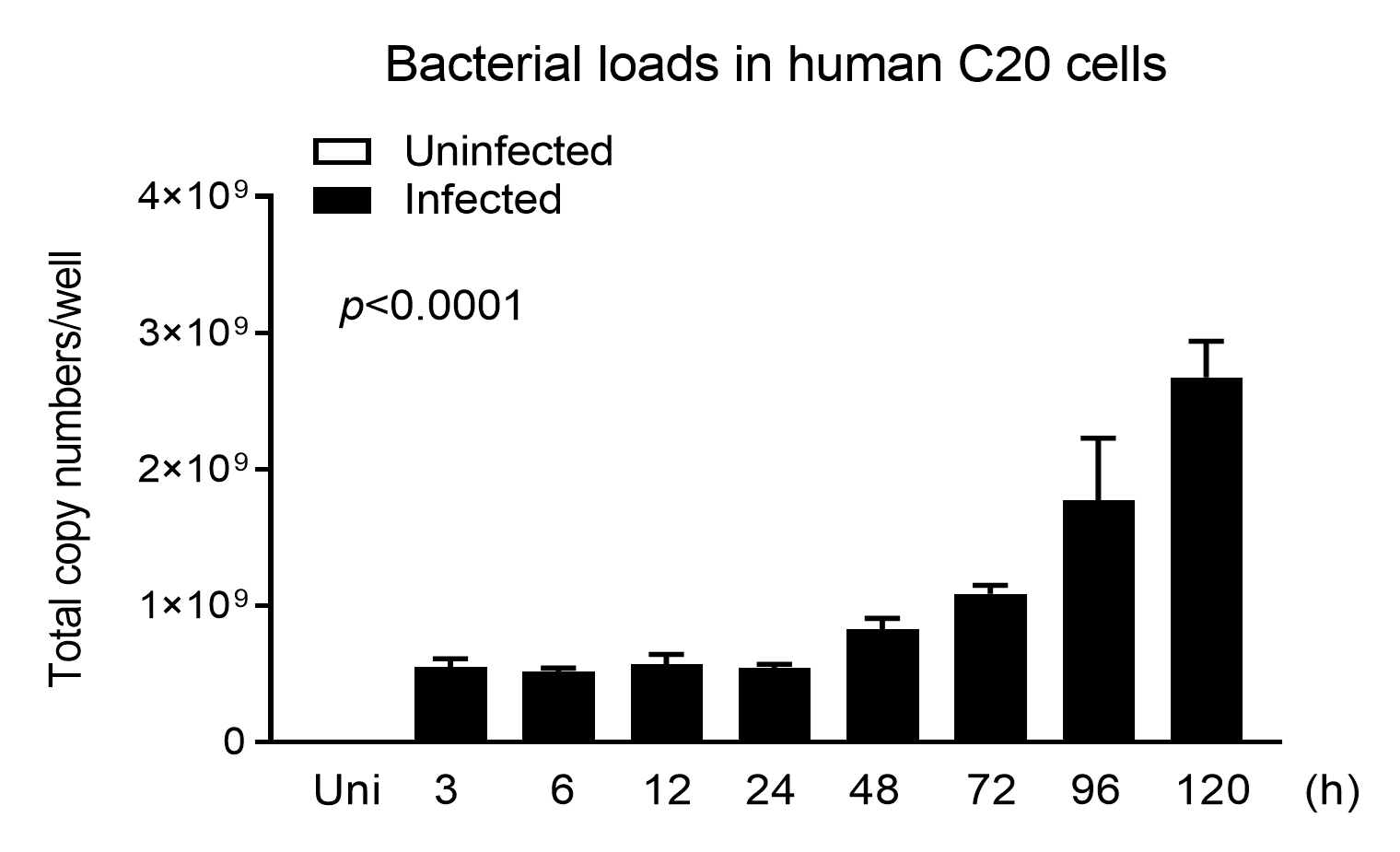

Supplement: Supplementary Figure 4 — Ot replication in a human C20 microglial cell line. C20 were infected with Ot in vitro (MOI 10) and uninfected cells were used as controls. Cells were harvested at indicated time points for bacterial burden measurement by qPCR. A one-way ANOVA with a Tukey’s multiple comparisons test was used for statistical analysis. This experiment was repeated twice independently with triplicate samples each time, and the data were shown from a single representative experiment. [file Image_4.tif]
