# Supplementary material for: Brain transcriptomics reveal the activation of neuroinflammation pathways during acute Orientia tsutsugamushi infection in mice
Source: Front Immunol. 2023 Jun 22;14:1194881. doi: 10.3389/fimmu.2023.1194881 (PMC10326051; doi:10.3389/fimmu.2023.1194881)
Supplement: Supplementary file 5 [file Table_1.docx]

| **Supplementary table 1** | | | |
| --- | --- | --- | --- |
|  |  |  |  |

**Real-time PCR primers of genes**

**Mouse Forward (5’ to 3’) Reverse (5’ to 3’)**

Cxcl10 CCAAGTGCTGCCGTCATTTTC GGCTCGCAGGGATGATTTCAA

Casp4 ACAAACACCCTGACAAACCAC CACTGCGTTCAGCATTGTTAAA

Upp-1 ACAGGAACTGAAGCAAAGGAC GTTGAAATGGTAGAGCACGTCTT

Ch25h TGCTACAACGGTTCGGAGC AGAAGCCCACGTAAGTGATGAT

Selp CATCTGGTTCAGTGCTTTGATCT ACCCGTGAGTTATTCCATGAGT

LRG-1 TTGGCAGCATCAAGGAAGC CAGATGGACAGTGTCGGCA

Ccl2 TTAAAAACCTGGATCGGAACCAA GCATTAGCTTCAGATTTACGGGT

Sfn9 TCCTTAGTGGTGAAACGGTCT TCAGGTTGCTCACTCTGGTTG

Ptg2 TGAGCAACTATTCCAAACCAGC GCACGTAGTCTTCGATCACTATC

Mx1 GACCATAGGGGTCTTGACCAA AGACTTGCTCTTTCTGAAAAGCC

Mx2 GAGGCTCTTCAGAATGAGCAAA CTCTGCGGTCAGTCTCTCT

Isg20 TGGGCCTCAAAGGGTGAGT CGGGTCGGATGTACTTGTCATA

Isg15 GGTGTCCGTGACTAACTCCAT TGGAAAGGGTAAGACCGTCCT

Irf7 GAGACTGGCTATTGGGGGAG GACCGAAATGCTTCCAGGG

Irf1 ATGCCAATCACTCGAATGCG TTGTATCGGCCTGTGTGAATG

Oas3 TCTGGGGTCGCTAAACATCAC GATGACGAGTTCGACATCGGT

Rsad2 TGCTGGCTGAGAATAGCATTAGG GCTGAGTGCTGTTCCCATCT

Il1b GCAACTGTTCCTGAACTCAACT ATCTTTTGGGGTCCGTCAACT

TNFa CCCTCACACTCAGATCATCTTCT GCTACGACGTGGGCTACAG

GAPDH AGGTCGGTGTGAACGGATTTG TGTAGACCATGTAGTTGAGGTCA

**Human**

TNFa CCTCTCTCTAATCAGCCCTCTG GAGGACCTGGGAGTAGATGAG

IL1b ATGATGGCTTATTACAGTGGCAA GTCGGAGATTCGTAGCTGGA

Cxcl10 GTGGCATTCAAGGAGTACCTC TGATGGCCTTCGATTCTGGATT

GAPDH GGAGCGAGATCCCTCCAAAAT GGCTGTTGTCATACTTCTCATGG
